# FC-NIRS: A Functional Connectivity Analysis Tool for Near-Infrared Spectroscopy Data

**DOI:** 10.1155/2015/248724

**Published:** 2015-10-11

**Authors:** Jingping Xu, Xiangyu Liu, Jinrui Zhang, Zhen Li, Xindi Wang, Fang Fang, Haijing Niu

**Affiliations:** ^1^State Key Laboratory of Cognitive Neuroscience and Learning and IDG/McGovern Institute for Brain Research, Beijing Normal University, Beijing 100875, China; ^2^Center for Collaboration and Innovation in Brain and Learning Sciences, Beijing Normal University, Beijing 100875, China; ^3^Department of Neurology, Jilin Oil Field General Hospital, Jilin 138000, China; ^4^Circulating Department of Internal Medicine, Jilin Oil field General Hospital, Jilin 138000, China; ^5^Department of Psychology and Beijing Key Laboratory of Behavior and Mental Health, Peking University, Beijing 100871, China; ^6^Key Laboratory of Machine Perception, Ministry of Education, Peking University, Beijing 100871, China; ^7^Peking-Tsinghua Center for Life Sciences, Peking University, Beijing 100871, China; ^8^PKU-IDG/McGovern Institute for Brain Research, Peking University, Beijing 100871, China

## Abstract

Functional near-infrared spectroscopy (fNIRS), a promising noninvasive imaging technique, has recently become an increasingly popular tool in resting-state brain functional connectivity (FC) studies. However, the corresponding software packages for FC analysis are still lacking. To facilitate fNIRS-based human functional connectome studies, we developed a MATLAB software package called “functional connectivity analysis tool for near-infrared spectroscopy data” (FC-NIRS). This package includes the main functions of fNIRS data preprocessing, quality control, FC calculation, and network analysis. Because this software has a friendly graphical user interface (GUI), FC-NIRS allows researchers to perform data analysis in an easy, flexible, and quick way. Furthermore, FC-NIRS can accomplish batch processing during data processing and analysis, thereby greatly reducing the time cost of addressing a large number of datasets. Extensive experimental results using real human brain imaging confirm the viability of the toolbox. This novel toolbox is expected to substantially facilitate fNIRS-data-based human functional connectome studies.

## 1. Introduction

Functional near-infrared spectroscopy (fNIRS), a promising noninvasive imaging technique, has become an increasingly popular neuroimaging technique for brain function research in recent years [1–4]. This technique holds several advantages relative to functional magnetic resonance imaging (fMRI), namely, its instrument portability, high temporal sampling rate, and ability to perform long data acquisitions. Given the technique's specific strengths, fNIRS has been extensively used to localize brain activation during task states [5–10] and to identify functional connectivity (FC) during resting states in both normal and diseased populations [4].

For the study of resting-state fNIRS, one of its promising advances is the detection of resting-state FC [6, 11] and the characterization of the topological organization of the brain connectivity network [12]. The approaches of seed-based correlation analysis [6, 13, 14], whole-brain correlation analysis [15–17], and graph-theoretical topological analysis [12, 18] were primarily used to derive the resting-state FC and the brain network. Particularly, the seed correlation analysis calculates the resting-state FC by predefining a seed region and subsequently computing the temporal correlation between it and other regions. With seed-based correlation analysis, researchers have observed a strong FC between the bilateral sensorimotor [11, 13], auditory [13], and visual system [19] in adults and connectivity changes during the normal development of early infancy [5, 15] and in neurological disorders [19, 20]. Similarly, whole-brain correlation analysis calculates resting-state FC by examining the temporal correlation of a time series between any two measurement regions in the whole-brain range. Using this approach, Homae et al. [15] found that the cerebral FC changed dynamically in infants from several days old to months old. Additionally, using this method, Zhang et al. [17] showed that the dominant frequency of FC within one functional system in adults can be identified by introducing a priori anatomical information. In contrast to the previous two methods, the graph-theoretical topological analysis models the brain as a complex network and then provides a straightforward but powerful mathematical framework for characterizing the topological properties of the brain networks. With the graph-theoretical network analysis approach, our group constructed the first whole-brain FC network using fNIRS brain data [12] and found that the fNIRS brain network was topologically organized in a nontrivial fashion, for example, with a small-world and modular architecture. Furthermore, our study [18] also showed that the graph theory metrics of the fNIRS brain network were reliable across different scanning sessions. In summary, this progress in FC and network analysis demonstrates the increasing interests in the study of functional brain connectivity and network organization using the fNIRS technique.

As an emerging analysis strategy for fNIRS data and considering the complexity of FC and network analysis, it is necessary and important to develop an easy-to-use and efficient FC toolbox to facilitate fNIRS researchers. There are already several available fNIRS toolkits, such as Homer [21], NIRS-SPM [22], fOSA [23], NINPY [24], and NAP [25], which have greatly assisted with the preprocessing of fNIRS data and activation detection based on task data. However, it must be noted that toolkits for assessing the FC and network analysis of resting-state fNIRS data are still lacking.

In this study, to facilitate human functional connectome studies in the fNIRS field, we developed a MATLAB software package for fNIRS-based connectivity analysis, which is called FC-NIRS (functional connectivity analysis for near-infrared spectroscopy data) and can be downloaded freely from the website http://www.nitrc.org/projects/fcnirs/ as an open-source package. The package's functions include preprocessing, quality control, FC calculation, and network analysis. Although the fNIRS collection has a chainless feature, it also easily leads to motion-head artifacts. At the same time, there are usually many sources and detectors placed on the head that are used for a whole-brain network study, which thus inevitably lead to a loss of contact between certain optodes and the scalp. Therefore, the two primary types of noise (i.e., motion artifacts [26, 27] and a low signal-to-noise ratio due to poor contact between the optodes and scalp [24]) need to be checked before performing FC and network analysis.

## 2. Materials and Methods

### 2.1. Toolbox Development

#### 2.1.1. Development Environment

FC-NIRS was developed using MATLAB 2010b in a 64-bit Windows 7 environment. The data preprocessing and network analysis modules include two established packages, Hemodynamic Evoked Response (Homer) and Graph-Theoretical Network Analysis (Gretna), for fNIRS data processing and graph theory-based network analysis, respectively. This FC-NIRS toolbox has been successfully tested under different operating systems with MATLAB installed, such as Windows and Linux (Ubuntu and CentOS).

#### 2.1.2. Data Format

Currently, FC-NIRS can process two file types: one type is in the  .nirs format from the CW5/6 system (TechEn, Inc.) and the other type is in the  .csv format from ETG4000/7000 (Hitachi Inc.). In fact, the  .csv files can be easily transformed into.nirs files. Thus, in the following description, we mainly introduce the parameters that were included in  .nirs files. (1) *d*: this variable was the actual raw data that were variable. This variable had the dimensions of *〈*number of measurements*〉* × *〈*number of time points*〉*. The rows in *d* were mapped by the measurement list (the mL variable described below). The *d* variable could be complex (as in the case of sine-cosine demodulation for laser carrier frequencies). (2) *t*: this is a time variable describing the time length of the data collection. (3) SD: this variable was a structured variable that described the configuration of the probe (source-detector) geometry. Furthermore, during the stage of “processing,” a “.proc” file could be brought out for each participant after clicking the “RUN” button. The “.proc” file was a MATLAB file with four fields: (1) RawData, which recorded the raw optical density information as in the  .nirs file; (2) OD, which recorded optical density changes; (3) Conc, which recorded the time series of the relative concentration variations in oxyhemoglobin (HbO), deoxyhemoglobin (HbR), and the total hemoglobin (HbT); and (4) SD, which recorded the configurations of the sources, detectors, and measurement channels between the sources and detectors. The abundant information in the “.proc” file provided the convenience of processing batches for the subsequent FC calculation and network analysis in the toolbox.

#### 2.1.3. FC-NIRS Analysis Procedure

**Figure 1:**
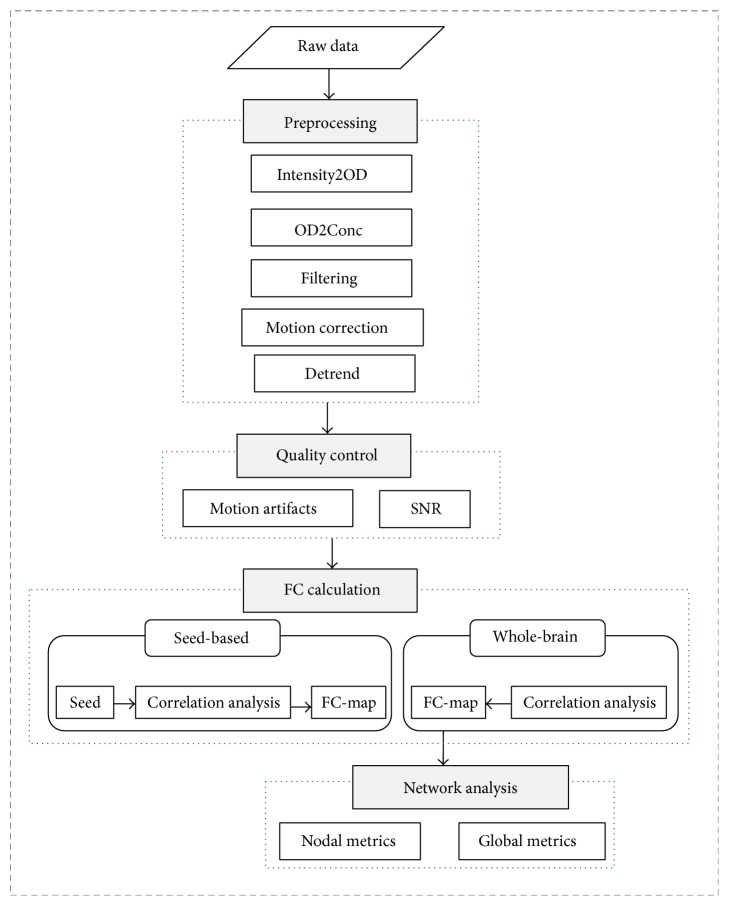
The main procedures for the processing of fNIRS datasets in FC-NIRS. The procedures contain four parts: (1) preprocessing, (2) quality control, (3) FC calculation, and (4) network analysis.

The main procedure of FC-NIRS is shown in Figure 1, and it included four main function modules: (1) preprocessing, (2) quality control, (3) FC calculation, and (4) network analysis.

### 2.2. Preprocessing

FC-NIRS provides a series of preprocessing methods in the panel of “Preprocessing Methods” (Figure 3), from which some methods can be selected and displayed in the panel of “Selected Methods.” Pressing the “≫” button selects a preprocessing method to the “Selected Methods” box, while pressing the “≪” button cancels the corresponding selected method. The “Up” and “Down” buttons were used to adjust the order of the selected methods. In the “input directory,” the users need to set a directory in advance to read the raw data. Similarly, in the “out directory,” the user also needs to provide an output directory to save the generated data. FC-NIRS also generated log files and kept track of the processing. After the operations and pressing the “RUN” button, FC-NIRS generated a “.proc” file for each subject in the output directory. For simplicity, FC-NIRS also provided some default preprocessing methods, which mainly included optical signal conversion, filtering, motion correction, and detrend. The details of the methods are as follows.

#### 2.2.1. Optical Signal Conversion

Similar to Homer software [21], the raw optical intensity was first normalized as the optical density (OD) to provide a relative (percent) concentration change by dividing by the mean of the intensity. Then, the OD data were further converted to HbO, HbR, and HbT based on the modified Beer-Lambert law [28].

#### 2.2.2. Filtering

FC-NIRS uses a band-pass filter with third-order Butterworth, zero-phase digital filtering for low-pass and fifth-order Butterworth, zero-phase digital filtering for high-pass to remove low-frequency noise, and physiological interference sources. The filtering range for the band-pass filter could be defined by the users themselves according to their study objectives. In the FC-NIRS toolbox, for convenience, we provided a default band-pass range from 0.01 to 0.1 Hz, which represents the frequency range of hemodynamic signals that are thought to emanate from spontaneous neural activity.

#### 2.2.3. Motion Correction

To reduce the motion-induced artifacts, FC-NIRS provided a spline interpolation method [29] and a correlation-based signal improvement (CBSI) method [30], respectively. Specifically, the spline interpolation method detected the motion-induced artifacts by calculating the moving standard deviation (MSD) within sliding time windows in a window length set by the user (default: 2 seconds). MSD values larger than the threshold defined by the user (default: five standard deviations away from the mean of the MSD) are regarded as artifacts. Next, the time series that represented the motion artifacts was further modeled via a cubic spline interpolation, which was subtracted from the original signal of the time series. The resulting signal was considered to be free of motion artifacts. By contrast, the CBSI was a type of channel-by-channel method that was based on the hypothesis that HbO and HbR should be negatively correlated during functional activation, while at the same time they should be more positively correlated when a motion artifact occurred. These approaches have demonstrated an improvement in the data quality through reducing motion artifacts [29, 30].

#### 2.2.4. Detrend

Previous studies demonstrated that systematic signal increases or decreases occurred over time due to long-term physiological shifts, with movement-related noise remaining. The linear trend is usually removed during fNIRS data preprocessing. Similar to the previous operation [4], FC-NIRS also estimated the linear trend with a least-square fit of a straight line and then subtracted it from the hemoglobin concentration signals.

### 2.3. Quality Control

To guarantee high quality data for the FC calculation and network analysis, a quality control module was designed to control the motion-induced artifacts and to lower the signal-to-noise ratio (SNR) that arose from poor contact between the optodes and scalp. For the head motion check, we calculated the sliding standard deviation of the time series of concentration signal to quantify the signal fluctuations within a series of sliding windows. The resulting time series of the sliding standard deviation was cut by a predefined threshold value *T*, and the values above the threshold value *T* were regarded as motion artifacts. FC-NIRS offers two types of display windows for the motion check at selected channels by clicking “Selected Channels” and the total channels by clicking “All Channels.” For the SNR check of the hemodynamic signal, FC-NIRS primarily examined the signal quality from the SNR optical intensity values and the signal correlation values in the concentration signal among all of the measurement channels. Because a low SNR value (equal to the mean signal intensity divided by the standard deviation of the signal intensity over time) in fNIRS measurements represents poor contact between the optodes and scalp, the quantification of the SNR values at all of the measurement channels allowed for the examination and identification of the measurement channels with poor quality. At the same time, we assumed that low SNR signals did not reflect real brain activity (similar to noise) and that, as a result, low SNR signals should have the smallest correlation with other measurement signals. Therefore, by performing a whole-brain correlation analysis, the correlation coefficients with nearly zeros between one measurement channel and the other channels were found to represent low SNR measurements. By double-checking the motion artifacts and the SNR, high quality data were identified for the subsequent FC calculation and network analysis.

### 2.4. FC Calculation

FC-NIRS provides two types of approaches for the FC calculation: a seed-based correlation method and a whole-brain correlation method. Specifically, the seed-based correlation method calculated FC by estimating the strength of the pairwise relationships between the seed regions and all of the other regions in the brain [4]. The whole-brain correlation analysis calculated FC by computing the connectivity strength between any two measurement channel pairs within the entire cerebral cortex. For each method, we provided three different correlation strategies, Pearson's correlation, Cross-correlation, and Spearman's correlation, based on three different hemoglobin concentration signals. The analysis can be performed at both the individual level and group level. For the group analysis, FC-NIRS offers several statistical correlation maps, such as the *R* map, the *Z* map, the *Z* to *R* map, and the *T* map. The *R* values in the *R* map represent the average of the correlation coefficients across participants; the *Z* values in the *Z* map represent the average of the *Z*-score of the FC; the *R* values in the *Z* to *R* map represent the correlation coefficients that were back-transformed from the average *Z* values; and the *t* values (uncorrected) in the *T* map represent the *t* statistical values after the one-sample *t*-test.

### 2.5. Network Analysis

FC-NIRS calculated the topological properties of the brain network based on a modern graph-theoretical approach [4, 12, 18]. The graph-theory approach is a straightforward and powerful tool for characterizing the topological architecture of brain networks. In the study of the fNIRS brain network, the channels are considered to be vertices and the FCs between any two channels are considered to be edges. Therefore, fNIRS data with *N* nodes forms an *N* × *N* correlation matrix and each value in the correlation matrix represents the FC strength. FC-NIRS calculated the global and local network metrics based on the Gretna package (http://www.nitrc.org/projects/gretna). Specifically, the global network metrics included small-world properties (clustering coefficient, characteristic path length, normalized clustering coefficient, and normalized characteristic path length), efficiency parameters (global and local efficiency), hierarchy, and modularity coefficients. These metrics were used to characterize the global topological organization of the whole-brain network. The nodal network metrics included the nodal degree, nodal efficiency, and nodal betweenness, which were used to examine the regional characteristics of the functional brain network. Of note, the diagonal elements in the correlation matrix were automatically set to “0” before network analysis. For more details about the graph metrics, see the report from Rubinov and Sporns [31].

### 2.6. Experimental Validation

#### 2.6.1. Subjects

Twenty-one healthy right-handed subjects (17 males and 4 females, aged 21 to 27 years) were recruited, and written informed consent was obtained from all of the participants prior to the experiment. This study was approved by the Institutional Review Board of Beijing Normal University Imaging Center for Brain Research. Of note, the data used in this study were obtained from a previous experiment that examined the test-retest reliability of the graph metrics of the resting-state fNIRS brain network [18].

#### 2.6.2. Data Acquisition

A continuous-wave (CW) near-infrared optical imaging system (CW6, TechEn Inc., MA, USA) was used to measure the variations of the HbO and HbR concentration. The system generated two wavelengths (690 and 830 nm) of near-infrared light and collected the hemoglobin-dependent signals at a sampling rate of 25 Hz. Twelve light sources (each with two wavelengths) and 24 detectors were designed to configure 46 measurement channels to allow for the whole brain (i.e., frontal, temporal, parietal, and occipital lobes) to be covered bilaterally (Figure 8(a)). The spatial separation between any adjacent source and detector pair was 3.2 cm. The positioning of the probes was set according to the international 10–20 system.

#### 2.6.3. Data Preprocessing

The default procedures were used for data processing and analysis. These procedures included the conversion of the optical density to the hemoglobin concentration, band-pass filtering, detrending, and motion correction using CBSI. For each method, default parameters were used for data preprocessing.

#### 2.6.4. Quality Control

We checked the quality of the fNIRS data by examining the motion artifacts and SNR. We discarded the data from 3 participants that had large motion artifacts and low SNRs.

#### 2.6.5. FC Calculation

We adopted the seed-based correlation method to calculate the FC map in which the seed region was located in the right visual cortex region. Pearson's correlation was adopted to measure the FC strength between the seed and the other brain regions.

#### 2.6.6. Network Analysis

Graph-theoretical approaches were used to characterize the topological properties of the fNIRS brain networks. For simplicity, we only examined the small-world feature to verify the validity of the network analysis in FC-NIRS.

## 3. Results

### 3.1. Toolbox Development

#### 3.1.1. Download and Installation

**Figure 2:**
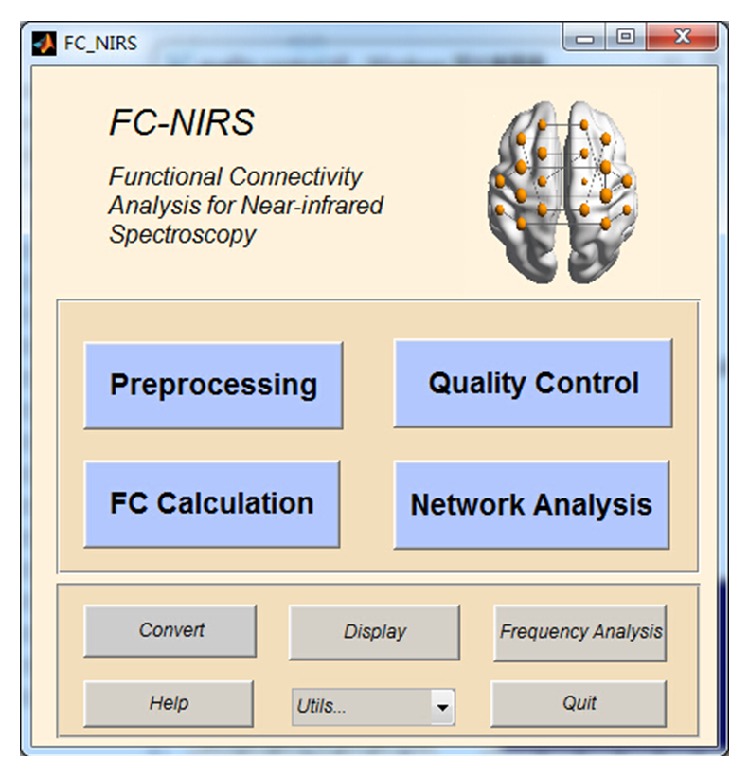
The main window of FC-NIRS. The four blue buttons are linked to four different functional modules, that is, preprocessing, quality control, FC calculation, and network analysis, which are shown in Figures 3, 4, 5, and 6, respectively.

**Figure 3:**
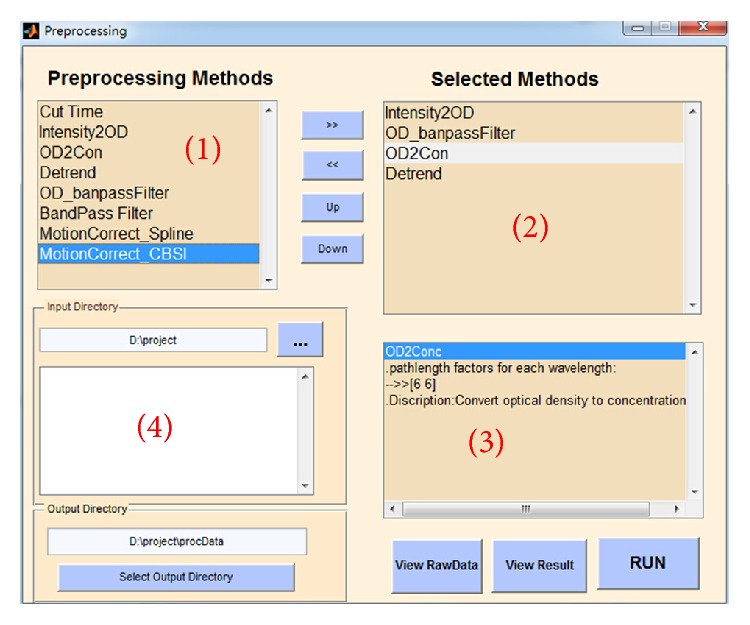
The preprocessing module of FC-NIRS. (1) Preprocessing methods provided by FC-NIRS; (2) preprocessing methods selected by users for data preprocessing; (3) parameter settings for selected methods; and (4) the input directory and output directory settings.

**Figure 4:**
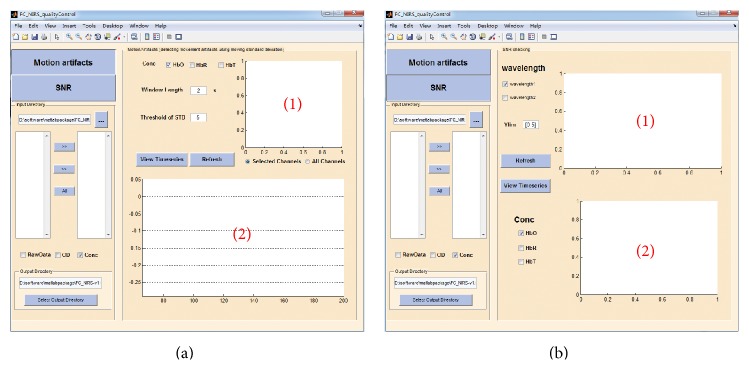
The quality control module of FC-NIRS. (a) The motion check, in which “(1)” shows the probe geometry for the imaging pad and “(2)” shows the time series of the moving standard deviation for the selected channels. (b) The SNR check, in which “(1)” shows the SNR values of all of the channels and “(2)” shows the correlation coefficients calculated from any two measurement channels.

**Figure 5:**
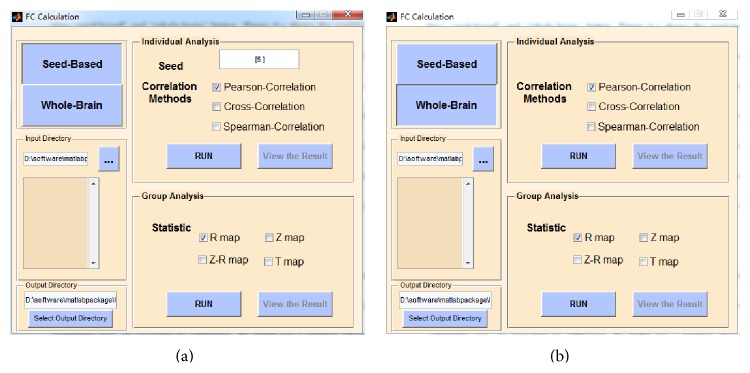
The FC calculation module of FC-NIRS. (a) The seed-based correlation analysis and (b) the whole-brain correlation analysis.

**Figure 6:**
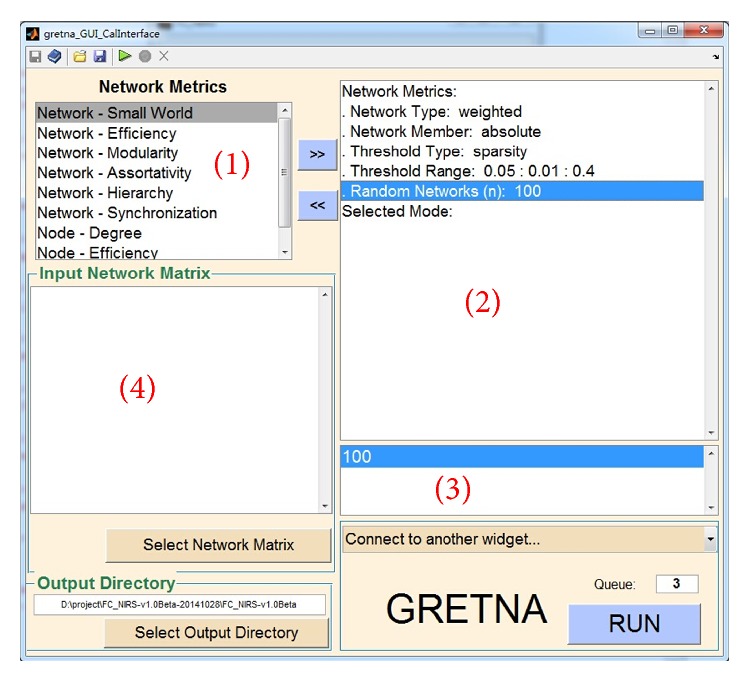
The network analysis module of FC-NIRS. Here, (1) shows the network metrics provided by FC-NIRS, (2) shows the network parameters (e.g., weight or binary network) and the selected network metrics, (3) shows the parameter settings for the selected network and network metrics, and (4) shows the input and output directory settings.

The FC-NIRS toolbox is an open-source package, and its source code is freely available at the website http://www.nitrc.org/projects/fcnirs/. The toolbox can run under both Windows and Linux operating systems with MATLAB installed. The installation of FC-NIRS is similar to that of most MATLAB software packages. To run the package, type “FC-NIRS” in the command window of MATLAB after adding the FC-NIRS folder in the MATLAB search path. To facilitate users who do not have MATLAB installed, we generated an actual binary executable file (FC_NIRS.exe) for windows users. As shown in Figure 2, the four buttons preprocessing (Figure 3), quality control (Figure 4), FC calculation (Figure 5), and network analysis (Figure 6) are linked to four primary functional modules. In addition, a user-friendly manual is available within the packages, which provides a detailed guide for using FC-NIRS.

#### 3.1.2. Quality Control

Figure 4(a) shows the GUI of the motion artifact check, which includes two panels that display the probe geometry (Figure 4(a)(1)) and the moving standard deviation of the concentration signals at the selected measurement channels (Figure 4(a)(2)). The window length and the threshold of the moving standard deviation in the panel can be set by clicking the “Refresh” button. FC-NIRS also offers a quick way to check the time series in all of the channels by clicking the “View TimeSeries” button.  Figure 4(b) shows the GUI of the SNR check, which also includes two panels that display the SNR values at all of the measurement channels (Figure 4(b)(1)) and the correlation matrix map calculated from the whole-brain time signals (Figure 4(b)(2)).

#### 3.1.3. FC Calculation

Seed-based (Figure 5(a)) and whole brain-based (Figure 5(b)) FC calculation methods can be selected by pressing the “Seed-based” or “Whole-brain” button. Figure 5(a) shows the GUI of the seed-based FC calculation. Within the panel, similar to the preprocessing procedure, the users must also set the input directory and the output directory in advance. To perform individual analyses, the user must input a seed channel and select a correlation method in advance. Afterward, the user can click the “RUN” button to obtain the individual analysis of the seed-based FC calculation. The user can view the correlation results by clicking the “View the Result” button. For the group analysis (Figure 5(a)), several different statistical maps (i.e., the correlation *R* map, the *Z* map, the *R*-*Z* map, and the *T* map) are generated by pressing the “RUN” button within the group analysis panel. All of the results from both the individual and group analyses are saved in the output directory.

#### 3.1.4. Network Analysis

Figure 6 shows the GUI of the network analysis. The network analysis must use the results of the whole-brain FC analysis as its input data (Figure 6). The module enables users to calculate the global and nodal network properties in parallel. FC-NIRS has a number of advantages for network analysis: for example, (1) it can run jobs in parallel either on a single computer with multiple cores or in a computing cluster; (2) it can generate log files and keep track of the pipeline execution; and (3) the jobs will run in the background and FC-NIRS and MATLAB can be closed after clicking the “RUN” button.

### 3.2. Validation

#### 3.2.1. Quality Control

**Figure 7:**
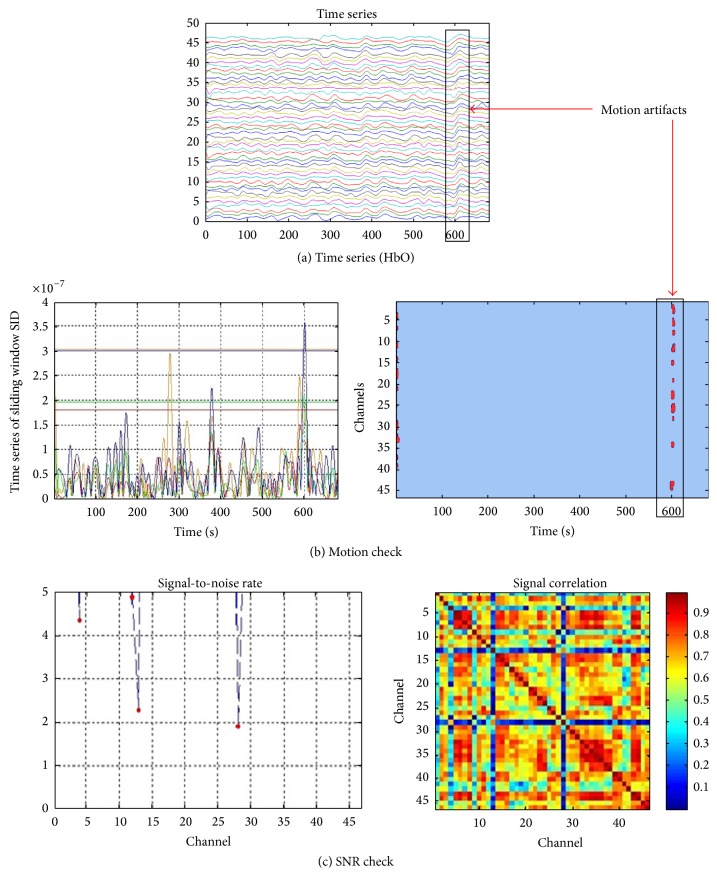
The experimental example for quality control of human brain data. (a) The time series of the hemoglobin concentration signals. A large fluctuation can be found at approximately 600 seconds. (b) The motion artifacts check. The window length is set to 2 s. The threshold is set to 5, which means that the values larger than five standard deviations from the mean are considered to be motion artifacts. (c) The SNR check. Channel 13 and channel 28 both have very low SNRs, which are computed as the mean signal intensity divided by the SD of the signal intensity over time compared with the other channels, and the corresponding signal correlation is very low. The low SNR of two channels can be caused by poor contact between the optodes and the scalp.

Figure 7(a) shows an example of the hemoglobin time series from a subject in which clear head motion was visually observed. With the motion detection method (i.e., the moving standard deviation method), the head motion was identified (Figure 7(b)). Notably, the moving window length was two seconds and the threshold for head motion was five times larger than the standard deviation of the moving standard deviation. In contrast, for the SNR check of the participant data, the SNR values of all of the channels are shown in Figure 7(c), from which two obviously low SNR channels (i.e., channels 13 and 28) were identified. Similarly, based on the whole-brain signal correlation analysis, we also identified two channels that had much lower correlation coefficients between them and the other measurement channels. The low correlation could be attributed to poor contact between the optodes and scalp. Based on this analysis, the subject data must be removed from the group data.

#### 3.2.2. FC Analysis

**Figure 8:**
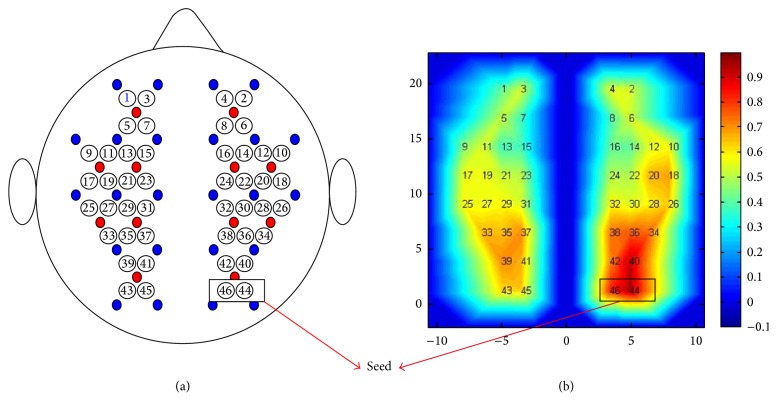
The results of the seed-based FC map. (a) The arrangement of the whole-brain 46-measurement channels on a brain template. (b) FC map (*R* map). Two channels in the black rectangle are used for the seed regions.

Adopting the seed-correlation method (the seed point in the right visual cortex, Figure 8(a)), we observed bilateral FC patterns between the left and right visual regions (Figure 8(b)). The results are consistent with those of previous fNIRS investigations [11].

#### 3.2.3. Network Analysis

**Figure 9:**
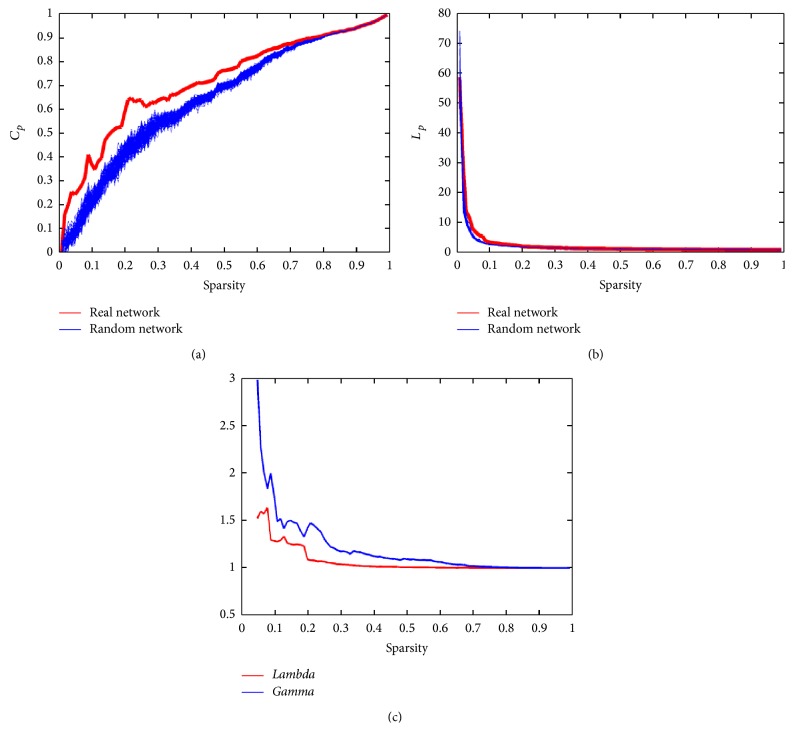
The results of the network analysis. (a) *C*_*p*_ is the clustering coefficient; the red line is the *C*_*p*_ of the real network, and the blue lines are the *C*_*p*_ of the random network. (b) *L*_*p*_ is the characteristic path length; the red line is the *L*_*p*_ of the real network, and the blue lines are the *L*_*p*_ of the random network. (c)* Lambda* is the normalized clustering coefficient, which is plotted with a red line;* Gamma* is the normalized characteristic path length, which is plotted with a blue line.

Figure 9 shows the small-world properties (clustering coefficients and characteristic path lengths) of the fNIRS brain network. Compared to matched random networks, we found that the real brain network has larger clustering coefficients, *C*_*p*_, and numerically similar characteristic path lengths, *L*_*p*_. These results are typical features of small-world topology and are also similar to our previous results [12].

## 4. Discussion

In this study, we developed a MATLAB software package called FC-NIRS for analyzing brain FC and networks from fNIRS data. The toolbox includes several major functions, such as data preprocessing, quality control, FC calculation, and network topological analysis. Furthermore, FC-NIRS allows for individual analysis of a group of participants using the module “quality control” and batch processing analysis in the other modules (i.e., preprocessing, FC calculation, and network analysis), which facilitates the calculation of the FC and network matrices with both high quality control and high efficiency using FC-NIRS.

Notably, different software packages for fNIRS data processing and analysis exist, for example, the widely used Homer [21] and the recently developed NIRS-SPM [22] packages. Of note, these two tools have a focus on activation detection during task states. Different from the Homer or NIRS-SPM, our FC-NIRS tool primarily aims at FC calculation and network analysis during task free or resting states. This work fills a gap in brain network research, specifically, the previous lack of software for fNIRS data. Furthermore, FC-NIRS provides different file formats (e.g.,  .nirs from TechEn, Inc., and  .csv from Hitachi Inc.) for data importation and analysis, which facilitates the use of different fNIRS imaging systems for FC calculation and network analysis. The ability to convert  .csv files to  .nirs files is also provided by FC-NIRS.

In its FC calculation and network analysis, FC-NIRS has great applicability in the field of connectivity neuroscience. For example, this tool can be applied to fNIRS datasets that are collected to study different connectivity hypotheses. Additionally, the evaluation of the effect of fNIRS imaging duration, correlation strategies, and frequency-band selection on the graphic metrics of brain network can be easily tested using FC-NIRS. Currently, the concept of “connectome” [32, 33] has been proposed to advance our understanding of comprehensively mapping and analyzing brain FC and networks [33], and fNIRS has been considered to be a promising technique for the study of functional connectome [4], especially during early childhood development and in unconscious patients. To handle the fNIRS-based connectome dataset, FC-NIRS has unique advantages, as it can process a large number of datasets in an efficient manner because of its batch processing strategies. Therefore, FC-NIRS can potentially make contributions to the study of the functional brain connectome in the future.

In the present study, we applied FC-NIRS to generate results for testing the resting-state FC in the bilateral visual cortex as well as network topological analysis at the whole-brain scale. Symmetrical FC was found in the bilateral visual system, which is highly compatible with previous findings [11, 34]. In addition, significant small-world features were observed in the whole-brain fNIRS network, which is also highly consistent with our previous results using the same dataset [12]. The present findings confirm the usability and validity of the FC-NIRS package.

In summary, FC-NIRS can facilitate and simplify the FC and network analysis in fNIRS-related studies and can provide optional FC definitions and network topological measures. However, some improvements in the software, such as providing statistical analysis for multiple comparisons and corrections for *T*-maps, are still required. Because FC-NIRS includes an extendable design framework, new functions for statistical analysis or new utilities can and will be added to future releases of the software.

## References

[1] Ferrari M., Quaresima V. (2012). A brief review on the history of human functional near-infrared spectroscopy (fNIRS) development and fields of application. *NeuroImage*.

[2] Scholkmann F., Kleiser S., Metz A. J. (2014). A review on continuous wave functional near-infrared spectroscopy and imaging instrumentation and methodology. *NeuroImage*.

[3] Tak S., Ye J. C. (2014). Statistical analysis of fNIRS data: a comprehensive review. *NeuroImage*.

[4] Niu H., He Y. (2014). Resting-state functional brain connectivity: lessons from functional near-infrared spectroscopy. *The Neuroscientist*.

[5] Nakano T., Watanabe H., Homae F., Taga G. (2009). Prefrontal cortical involvement in young infants' analysis of novelty. *Cerebral Cortex*.

[6] Niu H., Khadka S., Tian F. (2011). Resting-state functional connectivity assessed with two diffuse optical tomographic systems. *Journal of Biomedical Optics*.

[7] Taga G., Asakawa K., Maki A., Konishi Y., Koizumi H. (2003). Brain imaging in awake infants by near-infrared optical topography. *Proceedings of the National Academy of Sciences of the United States of America*.

[8] Gervain J., Macagno F., Cogoi S., Peña M., Mehler J. (2008). The neonate brain detects speech structure. *Proceedings of the National Academy of Sciences of the United States of America*.

[9] Sugiura L., Ojima S., Matsuba-Kurita H. (2011). Sound to language: different cortical processing for first and second languages in elementary school children as revealed by a large-scale study using fNIRS. *Cerebral Cortex*.

[10] Zeff B. W., White B. R., Dehghani H., Schlaggar B. L., Culver J. P. (2007). Retinotopic mapping of adult human visual cortex with high-density diffuse optical tomography. *Proceedings of the National Academy of Sciences of the United States of America*.

[11] White B. R., Snyder A. Z., Cohen A. L. (2009). Resting-state functional connectivity in the human brain revealed with diffuse optical tomography. *NeuroImage*.

[12] Niu H., Wang J., Zhao T., Shu N., He Y. (2012). Revealing topological organization of human brain functional networks with resting-state functional near infrared spectroscopy. *PLoS ONE*.

[13] Lu C.-M., Zhang Y.-J., Biswal B. B., Zang Y.-F., Peng D.-L., Zhu C.-Z. (2010). Use of fNIRS to assess resting state functional connectivity. *Journal of Neuroscience Methods*.

[14] Zhang Y.-J., Lu C.-M., Biswal B. B., Zang Y.-F., Peng D.-L., Zhu C.-Z. (2010). Detecting resting-state functional connectivity in the language system using functional near-infrared spectroscopy. *Journal of Biomedical Optics*.

[15] Homae F., Watanabe H., Otobe T. (2010). Development of global cortical networks in early infancy. *The Journal of Neuroscience*.

[16] Sasai S., Homae F., Watanabe H., Taga G. (2011). Frequency-specific functional connectivity in the brain during resting state revealed by NIRS. *NeuroImage*.

[17] Zhang Y.-J., Duan L., Zhang H., Biswal B. B., Lu C.-M., Zhu C.-Z. (2012). Determination of dominant frequency of resting-state brain interaction within one functional system. *PLoS ONE*.

[18] Niu H., Li Z., Liao X. (2013). Test-retest reliability of graph metrics in functional brain networks: a resting-state fNIRS study. *PLoS ONE*.

[19] White B. R., Liao S. M., Ferradal S. L., Inder T. E., Culver J. P. (2012). Bedside optical imaging of occipital resting-state functional connectivity in neonates. *NeuroImage*.

[20] Imai M., Watanabe H., Yasui K. (2014). Functional connectivity of the cortex of term and preterm infants and infants with Down's syndrome. *NeuroImage*.

[21] Huppert T. J., Diamond S. G., Franceschini M. A., Boas D. A. (2009). HomER: a review of time-series analysis methods for near-infrared spectroscopy of the brain. *Applied Optics*.

[22] Ye J. C., Tak S., Jang K. E., Jung J., Jang J. (2009). NIRS-SPM: statistical parametric mapping for near-infrared spectroscopy. *NeuroImage*.

[23] Koh P. H., Glaser D. E., Flandin G. (2007). Functional optical signal analysis: a software tool for near-infrared spectroscopy data processing incorporating statistical parametric mapping. *Journal of Biomedical Optics*.

[24] Strangman G. E., Zhang Q., Zeffiro T. A. (2009). Near-infrared neuroimaging with NinPy. *Frontiers in Neuroinformatics*.

[25] Fekete T., Rubin D., Carlson J. M., Mujica-Parodi L. R. (2011). The nirs analysis package: noise reduction and statistical inference. *PLoS ONE*.

[26] Molavi B., Dumont G. A. (2012). Wavelet-based motion artifact removal for functional near-infrared spectroscopy. *Physiological Measurement*.

[27] Piper S. K., Krueger A., Koch S. P. (2014). A wearable multi-channel fNIRS system for brain imaging in freely moving subjects. *NeuroImage*.

[28] Kocsis L., Herman P., Eke A. (2006). The modified Beer-Lambert law revisited. *Physics in Medicine and Biology*.

[29] Scholkmann F., Spichtig S., Muehlemann T., Wolf M. (2010). How to detect and reduce movement artifacts in near-infrared imaging using moving standard deviation and spline interpolation. *Physiological Measurement*.

[30] Cui X., Bray S., Reiss A. L. (2010). Functional near infrared spectroscopy (NIRS) signal improvement based on negative correlation between oxygenated and deoxygenated hemoglobin dynamics. *NeuroImage*.

[31] Rubinov M., Sporns O. (2010). Complex network measures of brain connectivity: uses and interpretations. *NeuroImage*.

[32] Kelly C., Biswal B. B., Craddock R. C., Castellanos F. X., Milham M. P. (2012). Characterizing variation in the functional connectome: promise and pitfalls. *Trends in Cognitive Sciences*.

[33] Sporns O., Tononi G., Kötter R. (2005). The human connectome: a structural description of the human brain. *PLoS Computational Biology*.

[34] Zhang H., Zhang Y.-J., Lu C.-M., Ma S.-Y., Zang Y.-F., Zhu C.-Z. (2010). Functional connectivity as revealed by independent component analysis of resting-state fNIRS measurements. *NeuroImage*.

